# Goal-setting and volitional behavioural change: Results from a school meals intervention with vitamin-A biofortified sweetpotato in Nigeria

**DOI:** 10.1016/j.appet.2018.06.038

**Published:** 2018-10-01

**Authors:** C.J. Lagerkvist, J.J. Okello, S. Adekambi, N. Kwikiriza, P.E. Abidin, E.E. Carey

**Affiliations:** a Swedish University of Agricultural Sciences, Box 7013, 75007, Uppsala, Sweden; b International Potato Center, P.O. 29053, Kampala, Uganda; c International Potato Center, P.O. Box 3785, Fumesua, Kumasi, Ghana

**Keywords:** Goals, Instrumentality, Experientiality, Emotions, Micronutrients

## Abstract

Malnutrition, particularly vitamin A deficiency, is a major public health problem in many developing countries. This study investigated whether priming or self-generation of goals, or whether attention to instrumental or experiential goals together with use of a reminder condition or not, promotes dietary behaviour intentions and change. A set of 556 randomly selected children aged 7–12 in Osun state, Nigeria, participated in an four-week intervention and field experiment in which a meal based on orange-fleshed sweetpotato, rich in pro-vitamin A, was introduced on five occasions as a complement to the existing school meal. Baseline intentions, anticipated feelings and repeated measures of post-consumption and experience were assessed. The analyses included a generalised linear mixed model for consumption and a linear mixed model for feelings and experience. The results confirmed that attention to instrumental goals undermines goal pursuit, while a focus on experiential goals increases the persistence of pursuit. Priming of experiential goals should be recommended, especially because this approach evokes positive feelings after eating. There was no evidence of an effect from repeated pairing of goals with the school meal, but use of planning by stating intentions increased the amount eaten. These results have implications for how school meals programmes should be designed to better align personal motivation with behavioural change in relation to dietary health.

## Highlights

• The impact of goals for dietary change among schoolchildren was studied.
• Instrumental goals undermined goal pursuit, but did not negatively affect feelings.
• Experiential goals increased the persistence of pursuit.
• Use of reminders was ineffective, but stating intentions increased the amount eaten.
• Priming of experiential goals is recommended in school meals programmes.

## 1 Introduction

Malnutrition is a major global challenge, particularly in developing countries. Recent efforts to fight malnutrition have targeted micronutrient deficiency, including vitamin A, zinc and iron. Besides being identified as a health problem, malnutrition has adverse effects on children's cognitive abilities, leading to poor school attendance and impairment of individual development (Olusanya, 2010). In Nigeria, recent statistics indicate that national malnutrition rate is about 30% and that malnutrition affects around 450,000 children below the age of five every year (Federal Ministry of Health, 2012).

There has been an increasing quest for theory-based nutrition and health education programmes that target behavioural changes, because such interventions are more effective than those lacking a theoretical base (Glanz & Bishop, 2010). School meals programmes that involve alteration of choice architecture to promote, or nudge, healthier eating habits have been shown to increase overall consumption of healthier foods (Da Costa, Møller, Bom Frøst, & Olsen, 2017). The literature on children's eating behaviours, and how to change these, shows that key cognitive constructs such as nutrition knowledge, social modelling and health considerations explain variations in eating behaviours (Ball et al., 2009). Similarly, programmes developed to increase children's knowledge and self-efficacy in relation to healthy nutritional practices have been shown to result in increased consumption of fruit and vegetables (Tuuri et al., 2009).

Many nutritional education programmes have been designed to include extrinsic incentives, e.g. by use of promotional messages or certain rewards. Previous research supports interventions that draw on extrinsic motivation, including use of rewards in the form of gifts, for goal attainments to promote certain behaviours (Grubliauskiene, Verhoeven, & Dewitte, 2012; Horne et al., 2011). It also supports the use of motivational interviewing to support goal selection in health promotion programmes (Draxten, Flatten & Fulkerson, 2016). These studies suggest that children's participation in the creation of promotional materials enhances the effectiveness of such campaigns and commitment to dietary change (Gustafson, Abbey, & Heelan, 2017).

However, there has been little research on the effects of specific goal setting in generating volitional, and maintained, dietary change among children. A study by Cullen et al. (2004) reported modest effects from giving children goals related to change in fruit, juice and vegetable consumption. Goal-setting theory defines goals in relation to their motivational meaning as self-generated desired end-states (Kruglanski et al., 2002). Therefore, goal setting is relevant as a behavioural change mechanism to combat malnutrition within school meals programmes because, as noted by Jensen et al. (2012, pp. 1019–1020), “people do not choose nutrients, they choose foods that contain nutrients”. Indeed, goal setting has been found to be effective in improving dietary change and intake of fruit and vegetables (Cullen, Watson, Zakeri, Baranowski, & Baranowski, 2007; White & Skinner, 1988). Little, however, is known about the specific contribution of the goal-setting process to these outcomes.

The specific objective of this study was therefore to analyse how specific goal setting promoted dietary behaviour change in relation to consumption of pro-vitamin A rich orange-fleshed sweetpotato (OFSP) among schoolchildren in Nigeria. OFSP is widely grown throughout sub-Saharan Africa for its leafy green and fleshy storage roots. While not the most commonly cultivated form in many places, OFSP is a rich source of dietary pro-vitamin A and can contribute very effectively to combating vitamin A deficiency (Hotz et al., 2012; Low, Mwanga, Andrade, Carey, & Ball, 2017).

### 1.1 Theoretical background and the present study

Nutritional education programmes are typically developed to communicate certain health or dietary benefits. Hence, there is a certain element of priming of the goals that the intervention seeks to promote, and ultimately achieve, in the form of behavioural change among the beneficiaries. However, goals are mostly self-generated and need to be considered attainable in order for people to commit to them (Bandura, 1997). Jin, Xu, and Zhang (2015) noted that people tend to focus on the properties of the goal when assessing the likelihood of attainment, and then fail to consider how their commitment will change during pursuit of the goal. Priming serves to facilitate and activate awareness, reasoning and interpretation of the goal properties. It has the potential risk that the depth and content of the communication as received and transformation of the information into goal commitment cannot be ascertained ex ante. Self-generated goals, on the other hand, are likely to be more aligned with attainability provided by personal control over the choice of motivational factors. However, presence of subjective difficulty, or inability to identify goals, can undermine the behavioural effects and influence the observed results (e.g. Schwartz et al., 1991).

Recent work in the goal-setting literature, shows that the impact of attending to goals depends on whether the benefits an individual gets from undertaking a certain activity are based on external or internal needs with further impacts on motivation and pursuit of the activity (Fishbach & Choi, 2012). In particular, an activity may be either extrinsically motivated, meaning that there are external benefits and rewards (instrumental goals) from performing the activity, or it may be motivated by intrinsic experiences (experiential goals). Furthermore, Fishbach and Choi (2012) indicated that the readiness for behavioural change increased with attention to instrumental goals, whereas actual pursuit increased with attention to experiential goals. Interestingly, a focus on instrumental goals also negatively affected feelings associated with goal fulfilment. This was because attention to such goals was perceived as more effortful and less pleasant to pursue.

It is relevant to examine the differences in behavioural outcome of goal-setting in a nutrition and health education perspective, because eating OFSP as part of a well-balanced school meal can be an instrumental activity that serves some desired extrinsic end-state or reward (i.e. goal). Such could include being health-improving, filling and satiating. It can also be an experiential activity with the focus on the meal itself, with intrinsic and experiential outcomes related to anticipated tastiness, joyfulness etc.

In this study, we extended the approach taken by Fishbach and Choi (2012) and investigated the behavioural outcome differences between priming and self-generated instrumental and experiential goals. We predicted that the proportion of OFSP consumed increases with priming of goals (hypothesis H1). This is because external information about benefits from eating OFSP (i.e. priming) makes incentives more salient, and because less effort may be spent in self-generating the goals. In comparison with self-generated goals, we predicted that the effect of priming is more prominent for those considering experiential goals (hypothesis H2). We, at the same time, predicted that priming of goals relates to more positive feelings (hypothesis H3a), although it is reasonable to expect that this effect is more prominent when the priming is directed to experiential goals (hypothesis H3b).

Portraying the meal as instrumental (e.g. healthy) instead of experiential (e.g. joyful) has motivational consequences and can therefore be expected to impact upon people's judgment and behaviour. Therefore, following Fishbach and Choi (2012) we assessed whether mere attention to instrumental goals *compared with* attention to the experience of eating OFSP as part of the school meal was enough to affect behaviour. We predicted that when children are asked to focus on the instrumentality of eating micronutrients, they are likely to eat more OFSP than when they focus on the eating experience itself (hypothesis H4) because, as noted by Fishbach and Choi (2012, p. 100), “introducing external incentives reduces an activity's intrinsic value”.

Goal setting as a motivational force relates to commitment and persistence through cognitive and emotional factors (Kopetz, Kruglanski, Arens, Etkin, & Johnson, 2012). Therefore, with regard to behavioural maintenance (i.e. continued eating of OFSP over time), we predicted that compared with a focus on the experience of eating OFSP (as part of the meals), a focus on the instrumentality of eating OFSP decreases the proportion of OFSP eaten as part of the school meal over time (hypothesis H5). Paying attention to instrumental goals is less likely to be a default state of mind and such a focus may require successively more effort because higher effort is required in pursuits that are less pleasant, with the potential to reduce goal pursuit. On the other hand, an action that focuses on the positive experience when eating the school meal may reinforce the motivation to pursue the activity. Correspondingly we predicted that a focus on instrumentality undermines persistence by negatively affecting feelings related to eating OFSP as part of the school meal (hypothesis H6).

Moreover, we explored the behavioural consequences of planned intake as a function of goal type. We predicted that planning as evidenced by behavioural intentions to eat the OFSP meal contributes to eating relatively more OFSP. This is because the benefits from the activity become more salient through the attention to goals that the planning provides (hypothesis H7).

The second way in which we extended the approach of Fishbach and Choi (2012) was to introduce a reminder component as part of the goal-setting intervention. This extension acknowledges the need for planning and behavioural maintenance in the post-intentional phases (Gollwitzer, 1991). Existing research shows that use of repeated exposure and associative conditioning relates to positive change in children's food acceptance (e.g. Keller, 2014). Hence, as goals do not directly lead to a certain behaviour, the use of goal reminders serves to form a cognitive link between the relevant environmental cue (i.e. the school lunch) and the goal behaviour. This link can help support goal-consistent planning and implementation. Just as one can expect removal of external incentives to make an individual less likely to pursue a goal (Deci, 1971), it is possible that repeated pairing of goals with, for example, the school meal can generate a more enduring behavioural effect in relation to food choices and to the feelings derived from the target activity. We predicted that, irrespective of type of motivation (i.e. instrumental or experiential), introduction of the reminder tool would allow children to keep their focus on their goals, which will in turn increase the proportion of OFSP eaten (hypothesis H9).

## 2 Materials and methods

This study was conducted in accordance with the guidelines laid down in the Declaration of Helsinki. All procedures involving human subjects were approved by the State Government of Osun on 14 November 2016 (reference OESF/HP/167/100).

### 2.1 Study area

The study was performed in eight local government areas (LGAs) in Osun state, Nigeria, in November and December 2016. These LGAs were purposively selected because the International Potato Research Center and the collaborating NGO (Partnership for Child Development, PCD) had not yet introduced OFSP to their farmers or households. Hence, there was no reason to expect that children would be familiar to OFSP. All the LGAs selected were close to the state capital (Osogbo), to reduce the logistical and financial burdens of the research. Osun State runs the Osun Elementary School Feeding and Health Programme (O-MEALS) which provides one mid-morning school meal to grade 1–4 children in all 1382 public primary schools in the state. O-MEALS aims to ensure that: i) meal planning is adjusted to accommodate seasonality and local availability of ingredients, and ii) each pupil receives a balanced meal that provides a minimum of 33% of the Codex Alimentarius Commission standard for energy, key vitamins and nutrients, and iii) the pupils receive deworming treatment when necessary.

### 2.2 Data collection

For the purposes of the present study, 12 primary schools were randomly drawn from the set of primary schools in the eight selected LGAs. A total sample of 556 pupils (274 female, 282 male) in 3rd and 4th grades participated in the study. The pupils ate at their usual desks in their classroom and were served depending on the study treatment.

Two weeks prior to the study, all legal guardians gave their written informed consent to allow the research team to observe pupils' food choices and consumption in the school environment.

Three days prior to the start of the study, a school-based nutrition education intervention session was held in each classroom for one hour. The class teachers were trained by experts within the research team to present specially developed materials to inform the participants about: (a) the five food groups, the main nutrients and their roles in the human body; (b) the importance of a balanced diet; (c) the food pyramid; (d) healthy food choices; and (e) incorporating OFSP in foods as a source of vitamin A (see Supplementary material). The research team further ensured that the children would be able to freely select and eat OFSP as part of their school meals.

A survey questionnaire was administered to obtain baseline conditions before the nutrition education intervention session. It collected data about age, gender, number of siblings, whether parents lived together or not, breakfast routines, extent of physical activity and food poverty. Data was obtained from each class teacher on children's school performance, measured using a classification into high, median or low performance in school exams were also collected. Further recall data on food intake focusing number of times main food groups (fruit; vegetables; whole grains; protein) and other foods (fried; sweetened beverages; snacks) were consumed per day during a typical week were also collected.

Data were also collected on

*Breakfast routines*. This question inquired about the frequency of eating breakfast. In reference to a typical week, participants responded using one of five categories: none, 1–2 times, 3–4 times, 5–6 times and every day of the week.

*Food poverty*. Previous studies have indicated that food choices among vulnerable young people are associated with food poverty (Booth, 2006). One item inquired about how often the child goes to bed hungry because there is not enough food at home, using four categories: 0 = never; 1 = sometimes, 2 = often and 3 = always.

*Physical activity*. Previous studies have indicated that physical activity is a determinant of food choices (e.g. De Vet, de Ridder, & de Wit, 2011). Three items assessed the extent of physical activity. Children responded on a last seven-day recall question on engagement in the following items: “moderate intensity activities”, “vigorous activities” and “just walking to and from school, but none of the other activities”. Physical activity was coded based on whether the participant just walked to and from school ≥5 times per week, but did not participate in moderate to vigorous physical activities.

Furthermore, the survey included two psychosocial constructs, pertaining to self-efficacy and peer (family) influences:

*Self-efficacy*. Self-efficacy refers to the confidence in one's ability to perform a desired behaviour in different situations (Bandura, 1986). Previous studies suggest that self-efficacy can be assessed in primary school children in relation to consumption of fruit and vegetables (Cullen et al., 2003; Tuuri et al., 2009) and for physical activity and food choice (Dzewaltowski, Geller, Rosenkranz, & Karteroliotis, 2010). Children with higher self-efficacy are predicted to be more motivated to change, to put more effort into their attempts to change and to have a better chance of succeeding. Self-efficacy corresponding to a child's confidence that they can eat a healthy diet and be active was measured using a scale developed to gauge specific components of the intervention. Self-efficacy was measured using eight statements on a five-point Likert scale (Cronbach's alpha = 0.852, with anchors “I am sure I can't” and “I am sure I can” (e.g. “I can identify a healthy meal”, “I can choose a healthy meal at school”, “I can choose a healthy meal when my friends can not”). Six of the statements inquired about healthy eating at home or at school, while two statements related to physical activity. A factor analysis for the self-efficacy scale revealed that all items loaded >0.4 onto a single factor (Gorsuch, 1983). This was further supported by the Kaiser-Meyer-Olkin (KMO) statistics (0.84) and the Bartlett's test sign was <0.001.

*Family influences.* The literature indicates that peer modelling for healthy eating is a key influence on food choice (Da Costa et al., 2017; Van Duyn et al., 2001). Three items therefore inquired about the frequency with which a household member encouraged the child to eat more fruit and vegetables; involved the child in cooking/preparation of fruit and vegetables at home; and told the child that eating more fruit and vegetables is good for one's own health. Statements were asked in reference to a typical week and participants responded using a five-item scale from 0 = none to 4 = daily (Cronbach's alpha = 0.72). A “do not know” option was included, but only two participants used this option. Factor analysis revealed that all items loaded at least 0.54 onto a single factor with KMO = 0.654 and the Bartlett's test sign was <0.001.

The study then ran for a four-week period because of the objective to explore a utilitarian activity in which pursuit requires continued selection of the OFSP meal over time. The OFSP meals were prepared by caterers recruited and trained by O-MEALS. For each school, the OFSP meal was served alongside the standard O-MEALS menu and was offered on five occasions in each participating school over the duration of the study. This provision of choice was intended to generate an experience of autonomy and motivation to enhance healthier action. A meta-study by Patall, Cooper, and Robinson (2008) showed that children are more open to such autonomy than adults. The school canteen provides a trusted environment in which children can act in relation to their peers in a natural way, without parental pressure.

Depending on treatment, before eating all children were called by name to either receive their own goal card (reminder treatment) or a coloured card with just their name on it (no-reminder treatment) before eating. Cards differed in colour so that the participants had yellow cards, while non-participants had blue or red cards. The reason for the difference in colour was not communicated to the children, to keep the treatment anonymous. After eating, they left their card on their desk together with the two plates. This was done to facilitate recording of amounts of food eaten.

Children therefore ate from two plates to increase the salience of the two meals provided. Each serving of the OFSP meal was 500 g and the energy density was 100 Kcal/100 g (Haile & Getahun, 2018). We used round, flat disposable plates for OFSP, which was appropriate for our study since it matched the pie-chart format that was used by participants to report their intentions to eat the OFSP meal. The empty plate weighed 25 g. The disposable plates were re-used once, after being cleaned by the O-MEALS staff, and were replaced after two feeding days. The O-MEALS meal was served on the deep metal plates that are part of the O-MEALS programme. This difference in plates made it easier to distinguish between the two school meals during the recording of the observations. The average weight of the empty metal plates was 250 g. Each serving of O-MEALS food was 300 g. This portion size followed the Osun state recommendations based on age groups and the energy density was in the range of 379–530 Kcal, depending on type of meal served (for details see Agbon, Onabanjo, & Okeke, 2012). Based on experiences by the research team from a school feeding study in Ethiopia, the larger amount of the OFSP meal served was set with the expectation that children would not be able to finish the food and therefore to achieve discrimination between the two meal components.

### 2.3 Study design and study factors

**Fig. 1:**
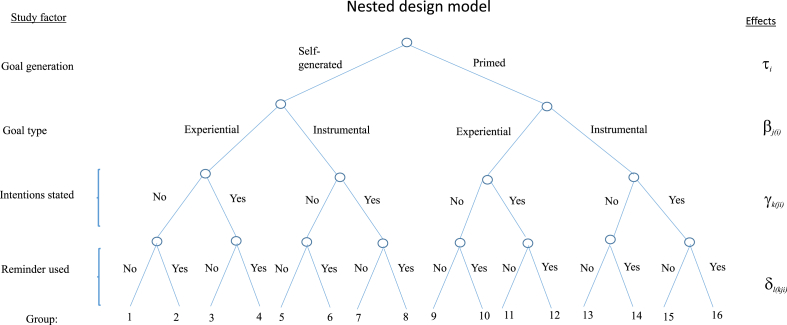
Schematic illustration of the study design.

The study design was nested because levels of a factor were unique to the levels of one or more other factors. The nested design was applied to match the theoretical considerations about the influence of the study factors, so as to allow for the measurement of the evidence for or against these considerations to be measured as accurately as possible. Fig. 1 presents a schematic overview of the five-level nested design (four study factors and the individual level (because of repeated measurement)). Level of the nested factor is denoted by the unique grouping factor (i.e. 1–16).

#### 2.3.1 Goal generation

**Fig. A1:**
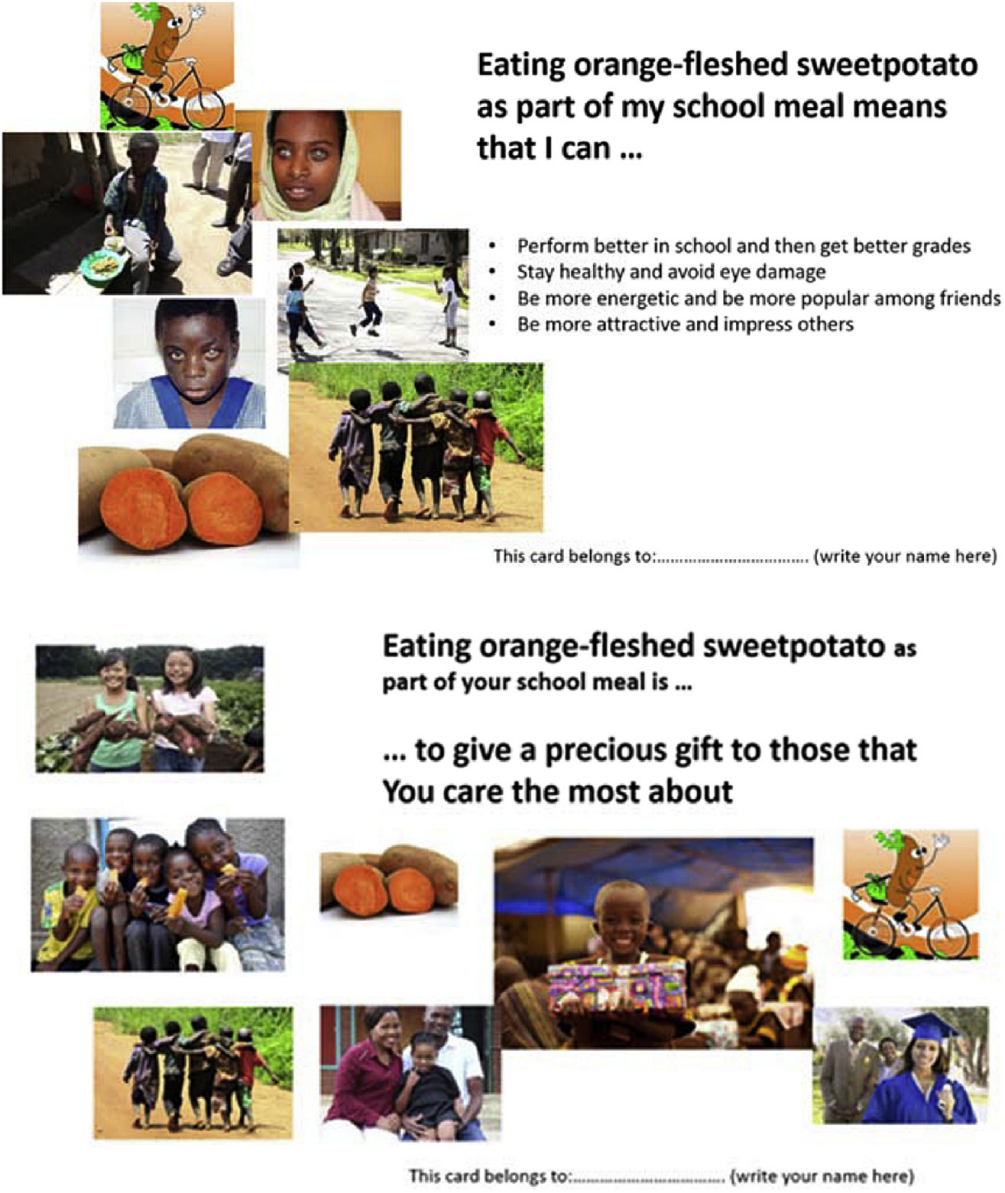
Material used for instrumental (upper panel) and experiential (lower panel) priming of orange-fleshed sweetpotato (OFSP) as part of the school meal.

Following the information session, to prime the goal of eating OFSP as part of the school meal we handed out a one-page information sheet (see Appendix, Figure A1). Participants in the instrumental treatment were presented with four extrinsic goals (Figure A1a), while participants in the experiential condition (based on the work by Dunn, Aknin, & Norton, 2008) saw a text (Figure A1b) that alluded to a typical intrinsic experience (i.e. giving to the most important others). Each of the primings included a set of images compatible with the verbal goals, since visual representations are processed differently from verbal messages (Biel, 1993). Recent findings on the use of pictures to study nutrition and hedonic perception in children show that pictures are helpful for the perception of healthiness (Varela & Salvador, 2014).

#### 2.3.2 Goal type

To obtain instrumental self-generated goals, participants were instructed to think about and formulate the goals they wanted to achieve by eating the OFSP school meal. To direct participants' attention to the experientiality of the meals, we instead instructed those in the experience group to think about and formulate their anticipated experiences from eating OFSP as part of the school meal. Participants noted their name, grade and own goals (space allowed for five goals) on a designated card. Children who were unable to write were assisted by the research assistants.

#### 2.3.3 Intentions stated

Next, to capture eating intention vs. pursuit, we asked those in the intention treatment just after the nutrition education intervention session (i.e. three days prior to the first serving) to indicate how much OFSP they expected to eat (using a share-of-plate figure) and to rate how their school meal would make them feel. For participants in the pursuit conditions we only measured the amount eaten. There is scant research on children's emotions related to food. A recent review by Laureati, Pagliarini, Toschi, and Monteleone (2015) suggests that children can be expected to perform most conventional consumer research tests. The study by De Pelsmaeker, Schouteten, and Gellynk (2013) reported that 8- to 13-year-old children were able to discriminate emotions by brands.

#### 2.3.4 Reminder used

On each observation day, those in the reminder treatment were provided with either their information sheet (card) in accordance with the goal type treatment or their own card (when goals were self-generated), and asked to read it before the school meal was provided. Those in the non-reminder treatment followed the same between-subject design, but for this group no card was shown prior to the lunch being served.

### 2.4 Measures

Two separate dependent variables were used in the subsequent analysis.

#### 2.4.1 Proportion of OFSP meal eaten

A linear ratio measure relating the quantity of the OFSP meal consumed to the total quantity of the school meal (OFSP meal plus O-MEALS meal) was obtained on each observation day and for each participant. The leftover amount of the OFSP and the standard O-MEALS meal was weighed on a balance after the school lunch (i.e. leftovers) and the difference relative to the serving size was recorded as the amount consumed by each pupil. The sensitivity of the balance was ±25 g.

#### 2.4.2 Feelings

Following the approach by Fishbach and Choi (2012), after eating each participant rated how their school meal made them feel. Feelings were captured using two measures on a five-point likert scale with anchors (1 = tired; 5 = refreshed, and 1 = sleepy; 5 = awake). To create an index of feelings related to the school lunch, the two items were averaged across participants (α = 0.742). A factor analysis for the feelings scale revealed that both items loaded >0.7 onto a single factor. This was further supported by the KMO statistics (0.50) and the Bartlett's test sign <0.001.

### 2.5 Data analysis

A mixed-model approach was taken to fit the nested design because such models are appropriate for modelling nested data sets (Schielzeth & Nakagawa, 2013). Mixed models estimate the standard errors more appropriately than classical linear models (Gelman, 2005). They reflect hierarchical and clustered data structures with individual observations at the lowest level (Bolker et al., 2009). In the approach taken, fixed factors were used for estimating the effect of the study factors, whereas random effects was used for controlling for correlated structure in the data. In particular, the nested factor (to account for the dependencies introduced by the experimental design) and the individuals (because of repeated measurement) were used as random factors. Variables/factors were coded as dummies for ease of interpretation of effects as differences between treatments.

A generalised linear mixed model (GLMM) with a binomial distribution and a logit link function fitted by maximum likelihood was used to determine the effect of goal setting on the proportion of OFSP meal eaten in response, because the data were proportional. The model is expressed as (Eq. (1): individual and time subscripts suppressed):

(1) $$y_{ijklm}=\mu +\tau_{i}+\beta_{j\left(i\right)}+\gamma_{k\left(ij\right)}+\delta_{l\left(ijk\right)}+\left(\tau \left(primed\right)\cdot \beta \left(experiential\right)\right)+\left(\beta \left(instrumental\right)\cdot time\right)+\varepsilon_{m\left(ijkl\right)}$$

where *μ* denotes the model intercept and $\varepsilon ∼\left(0,\sigma^{2}\right)$ is the iid error term. The order of the repeated measure (i.e. time) was modelled as a fixed effect because all levels of time occur with all participants. The adaptive Laplace approximation method was used to obtain a more accurate approximation for the maximum likelihood estimate of the model parameters, because the response variable was proportional to an expected value < 5 and because there was only one random variable (Bolker et al., 2009). Furthermore, the overdispersion statistic (i.e. combined residuals being much larger than the residual degrees of freedom) of the GLMM models was computed using the bmeco package for R version 2015.6–28 (R Core Team, 2015).

Next, a linear mixed model fit by maximum likelihood (LMM) was used to determine the effect of goal setting on feelings after eating (see Eq. (2)). This was appropriate, because the response variable was approximately normally distributed.

(2) $$y_{ijklm}=\mu +\tau_{i}+\beta_{j\left(i\right)}+\gamma_{k\left(ij\right)}+\delta_{l\left(ijk\right)}+\left(\tau \left(primed\right)\cdot \beta \left(experiential\right)\right)+\varepsilon_{m\left(ijkl\right)}$$

According to Barr, Levy, Scheepers, and Tily (2013), the detection of fixed effects from the study manipulations should also acknowledge the existence of participant random effects for the manipulations. Therefore, to test for the presence of idiosyncratic sensitivities to the study manipulations that might have an overall effect, three alternative random effects structures were examined in the estimation of Eqs. (1), (2). First, a nested random effect with participants clustered due to the group level (see Fig. 1) was modelled so that the intercept varied among groups and participants within groups (i.e. (1 | cell/id)). Two alternative models were then estimated with the random effects pertaining to either participants (i.e. (1 | id)) or groups (i.e.1 | groups), but without clustering. A set of likelihood-ratio tests was used to evaluate the evidence in the data for the extra complexity of the nested random effects relative to each of the simpler specifications (Christensen, 2015).

Next, following Baguley (2009), likelihood ratio tests were used to obtain effect sizes for the two models, for ease of interpretation. The null models were set so that the dependent variables were predicted only by their overall means. The set of variables included in the estimations were then expanded step-wise in the order given by Eqs. (1), (2).

The models were estimated in the lmer4 package (version 1.1–11) (Bates, Maechler, Bolker, & Walker, 2015) for R (R Development Team, 2015). Confidence intervals were calculated for the LMM model, to indicate the plausible range of values for the independent variables.

## 3 Results and discussion

### 3.1 Sample characteristics

The characteristics of the sample are shown in Table 1. Interviews with key informants revealed that class repetition is common in the Osun state, and in Nigeria in general, and is the main reason for the large variation in age of respondents. Subjects also had a large number of siblings, which was not unexpected. For example, Anyanwu (2014) reports large household sizes averaging 8.3 members in some states in Nigeria and attributes this to prevalence of poverty. This finding, based on Living Standards Survey data (Federal Ministry of Health, 2012), also explains why more than 50% of the respondents indicated that they had experienced food poverty sometimes, always or often during the week of the interview. The findings on prolonged physical activity were also in line with a priori expectations, as the majority of rural students in Nigeria, from which the study sample was drawn, walk to and from school every school day.

**Table 1:** Characteristics of the sample (n = 556, Nigeria).

Variable	Description	Min	Max	Mean	Std. Deviation
Gender	1 = male; 0 = female	0	1	0.5080	
Age^c^	Years	7	12	9.33	1.31
School performance	1 = high performance; 0 = average or low performance	0	1	0.3993	
Number of siblings		0	13	4.45	1.87
Being_oldest sibling	1 = yes; 0 = no	0	1	0.1907	
Parents live together	1 = yes; 0 = no	0	1	0.7059	
Breakfast routines	1 = 5–7 times/week; 0 = max 3–4 times per week	0	1	0.7790	
Food poverty	1 = sometimes/often/or always go to school hungry; 0 = never	0	1	0.5401	
Physical inactivity	1 = less active; 0 = more active	0	1	0.3280	
Self-efficacy	index (8 items)	1	5	3.448	1.29
Family pressure	index (3 items)	0	4	1.975	0.85

Note: ^a^ Median = 0.6452, 1st Qu. = 0.50, 3rd Qu. = 0.731, missing obs. = 227. ^b^ Missing obs. = 284. ^c^ The age of children varied because children start school at different ages and repeat school years.

Data on participants' food recall, food planning and family pressure are presented in Table 2. The results show that most of the respondents consumed most of the food groups 1 to 4 times per week. The finding that the majority who drank sweetened beverages did so only a few times per week was in line with our expectations. In rural conditions and under conditions of food poverty, it is to be expected that few families will devote money to buying non-essential foods such as sweetened drinks frequently. Furthermore, the finding that only a small minority of respondents (about one-third) were involved in food planning suggests that adult family members and school authorities make decisions about food choices. This finding is perhaps because of the feeling that the children (3rd and 4th graders) who made up the respondent population are still too young. The results also showed that family pressure regarding food choice was rated by the majority of the respondents as “sometimes”, indicating that most respondents came from families which were not overly pushy about foods eaten by their children. Again, this is due to the fact that the respondents were drawn from a rural population where getting food to eat seems to be a greater concern than the type of food eaten.

**Table 2:** Descriptive statistics on food recall, food planning and family pressure (%, n = 561, Nigeria).

Food recall^a^	Not at all	1-2 times	3-4 times	>5 times		
Eat fruit	0.02	0.298	0.365	0.335		
Eat vegetables	0.012	0.378	0.387	0.223		
Eat whole grains	0.011	0.342	0.371	0.276		
Eat lean protein	0.002	0.396	0.278	0.324		
Eat fried food	0.043	0.576	0.289	0.093		
Drink sweetened beverages	0.075	0.713	0.155	0.057		
Eat pastries, biscuits and sweets	0.061	0.497	0.209	0.234		
Food planning^b^	Not at all	1–2 days	3–4 days	5–6 days	7 days	
	0.169	0.369	0.125	0.057	0.28	
Family pressure	None	Once	Sometimes	Almost daily	Daily	Don't know
Encouragement^c^	0.10	0.166	0.487	0.159	0.086	0.004
Involvement^d^	0.119	0.112	0.515	0.125	0.125	0.004
Persuasion^e^	0.094	0.169	0.529	0.123	0.082	0.002

a Food recall based on the question “During a typical week: How often do you:”.

b During a typical week: How often do you help (or get involved) in planning meals at home?.

c During a typical week: How often has a member of your household encouraged you to eat more fruit and vegetables?.

d During a typical week: How often has a member of your household involved you in cooking/preparing fruit and vegetables for eating at home?.

e During a typical week: How often has a member of your household told you that eating more fruit and vegetables is good for your health?.

### 3.2 Goal-setting and behaviours

In total, 556 individuals participated in the study resulting in 2450 observations that were used for analysis. A total of 332 observations were incomplete (i.e. missing data) and were dropped from the analysis. Thus, the data were unbalanced, but the average number of observations per individual was still high (4.6). The average proportion of OFSP meals eaten was 0.58 (SD = 0.22) and the average score for the experienced feeling was 4.01 (SD = 1.07). Table A1 (Appendix) shows the amount of the two meal components, the proportion of the OFSP meal eaten and the number of observations and individuals per group of the nested study design.

Table 3 presents the results of the confirmatory hypothesis testing. Details of the estimates obtained using two mixed models for proportion of OFSP meal eaten and feelings experienced are presented in Tables A2 and A3 (Appendix), respectively. Tables A4 and A5 (Appendix) show the effect sizes for the study factors. The likelihood ratio tests of effect size measures showed strong support for the two model specifications, as opposed to the null models where the dependent variables were predicted by each of their overall means (Eq. (1): proportion of OFSP meal, df = 13, p < 0.001; Eq. (2): feelings, df = 9, p < 0.001). Furthermore, model comparisons of the three alternative random effects structures of the GLMM models of the proportion of OFSP meal eaten found no significant difference. Specifically, there were no difference between the simpler specification with a random intercept by participants (AIC = 851.4, df = 15) and the specification where the random variance for groups and participants was nested (AIC = 858.4, df = 16). Based on this finding, the results in Table A2 for the model specification with individual heterogeneity in response behaviour. However, in the feelings after eating model (Table A3), the nested random error structure (AIC = 6859.8, df = 13) was supported over the simpler model with just individual heterogeneity (AIC = 6886.6, df = 12).

**Table 3:** Overview of initial hypotheses and main findings.

Hypothesis	Proposition	Result
H1	Proportion of OFSP meal eaten is higher when goals are primed instead of self-generated	Not supported
H2	Priming is more effective when related to experiential goals rather than to instrumental goals	Supported
H3a	Priming of goals evokes more positive feelings than self-generated goals	Supported
H3b	The effect on feelings increases when priming refers to experiential goals	Not supported
H4	Proportion of OFSP meal eaten is higher when goals are instrumental rather than experiential	Supported
H5	Proportion of OFSP meal eaten declines more over time when goals are instrumental rather than experiential	Partly supported
H6	Instrumental goals evoke more negative feelings than experiential goals	Not supported
H7	Use of planning through stating intentions increases the proportion of the OFSP meal eaten	Supported
H8	Use of a reminder tool increases the proportion of the OFSP meal eaten	Not supported

There was no difference in the proportion of the OFSP meal eaten for the groups in which goals were self-generated or primed (H1). This finding suggests that the priming of goals, which may be inbuilt in the messages in pre-developed and standardised educational material, is not necessarily more effective than a focus on self-generated goals to obtain a behavioural change. Such messaging could be designed with or without the objective of alleviating subjective difficulty or inability to identify personal goals. However, our hypothesis (H2) that the effect of specific priming of experiential goals increases the proportion of OFSP eaten could not be rejected (odds ratio (OR) = 6.28). Moreover, children in the priming study had more positive feelings after eating the OFSP school-lunc than children with self-generated goals, supporting H3a. However, there was no support for an accentuated positive effect on feelings for those children who were primed with experiential goals. Hence, H3b was rejected.

Results further showed that a focus on instrumental goals had a substantial and positive effect on the proportion of OFSP school-lunch meal consumed (OR = 5.62). This finding confirmed our hypothesis (H4) that when children focus on the instrumentality of eating micronutrients, they are more likely to eat more OFSP meal than when they focus on the eating experience itself. Next, and relevant to behavioural persistence, there was little evidence to show that the proportion of the OFSP meal eaten was reduced over the duration of the study. Relevant to this finding is that there was strong evidence supporting the hypothesis that feelings of exposure to the OFSP meal became more positive over the duration of the study. Hence, the increase in positive experiences about eating the OFSP meal as part of their school lunch worked to offset the efforts related to goal fulfilment. However, the results of the goal × time interaction variable partly supported the prediction (H5) that the proportion of the OFSP meal eaten decreases over time when goals are instrumental. This finding suggests that this type of goal has a more negative influence on pursuit than experiential goals. This result therefore corroborates findings by Fishbach and Choi (2012) that instrumental goal priming undermines behavioural persistence. Moreover, there was no support for the hypothesis that a focus on instrumentality undermines behavioural change by negatively affecting the feelings related to eating OFSP as part of the school lunch meal (H6).

The results further supported the prediction (H7) that the proportion of the OFSP meal eaten increases for children who, at the time of formulating their goals, also state their intention to eat the OFSP meal (OR = 2.56). In addition, there was no evidence for the prediction that stating intentions affects feelings of eating the OFSP meal.

Moreover, pairing goals with stimuli through use of the cards, thus giving the children a chance to be reminded about their goals prior to eating, had no influence on the proportion of the OFSP meal eaten. Consequently, H8 was not supported. In addition, there was no support for the prediction that pairing the cards with the goals prior to eating the lunch contributed to less positive experiences. The OFSP meal appeared as part of a structured eating occasion and the result suggest presence of a conflict between giving in to some conventions for the lunches, on the one hand, and acting in support of the individual goals, on the other. In this case, the reminders were not effective enough to support implementation of intentions. Alternatively, the result could be interpreted as reactance, suggesting that the cards were not effective because children felt behaviourally impaired and wanted to regain their autonomy.

## 4 Conclusions and implications

Although the causes of child malnutrition are complex, interrelated and multidimensional (UNICEF, 1990), inadequate dietary intake (energy, protein, fat and micronutrients) and unhealthy eating behaviours due to inadequate household access to food, limited income and inadequate care of children are the most frequently cited contributing risk factors to child malnutrition (e.g. Linnemayr, Alderman, & Abdoulaye, 2008). From the plethora of possible solutions, schools have been identified as a key place to direct children's eating and health behaviours (Mullen & Shield, 2004).

Drawing on previous work on how thinking about goals undermines goal pursuit, this longitudinal study investigated whether specific goal setting promoted dietary behaviour change in relation to vitamin A deficiency among elementary schoolchildren in Nigeria. The results of this study have implications for future development of policy and practice on how school meals programmes can be designed to better align personal motivation with respect to dietary health.

First, a novel finding in this study is that goal priming or self-generation of goals had no significant difference in the proportion of the OFSP meal eaten depending on whether goals were primed or self-generated. Hence, personal control over choice of motivational factors did not undermine the behavioural effects, or influence the observed results, as could have been expected because the self-generation of goals requires mental effort. In fact, the results suggest that priming on experiential goals should be preferred over self-generated goals because this is concordant with the idea that the default state of mind is on intrinsic experiences.

Developing material to be used in school meals programmes to increase awareness and to induce behavioural change can be costly and comes with the problem that the information has to be processed at the level of each recipient for personal relevance. Nutrition information and school feeding programmes focusing on behavioural change typically include incentives in the form of associated explicit rewards for certain achievements. The educational and information material can therefore include explicit rewards, or invite beneficiaries to become involved in generating e.g. outcome expectancies from participation in the programme to which rewards are associated. Personal goals serve as a motivational force that enables identification and selection of appropriate means to the end desires. However, a key characteristic of a goal is that it is self-generated. It is therefore possible that programme rewards may be internalised as instrumental goals. Consequently, it can be argued that use of self-generated goals could be an interesting direction for increasing personal relevance and motivation for behavioural change in school meals programmes. A challenge would then be that children, in particular, would face difficulties setting more prospective, and therefore more motivational, intrinsic goals.

Second, the type of goal set was relevant to the behavioural outcome. Instrumental goals were more effective than experiential goals. However, there was a conflict between goal type and behavioural persistence: instrumental goals undermined behavioural persistence. This corroborates the findings by Fishbach and Choi (2012). Furthermore, the reduced persistence from using instrumental goals could not be related to such goals evoking more negative feelings, as suggested by previous research (Fishbach & Choi, 2012). Our findings suggest therefore that it is the type of incentives that matters, and not the emotional effects after eating. Hence, emotions are probably more related to the eating situation than to the type of goals set for the behaviour. Based on the results, it is advisable to be careful when using explicit rewards to influence behaviour change. This findings is not totally unexpected from the perspective of the motivational literature. However, more research is needed to address how to develop incentives that become internalised as, and aligned with, experiential goals. For example, interventions such as the promotion of OFSP among schoolchildren should shift attention from making health benefits explicit to targeting and communicating the experiential benefits related to the potential health benefits. This finding also has implications for product development. It implies that crop breeding should emphasise traits that enhance positive experiences when eating OFSP foods.

Third, we found that directing children to state their intention to eat the OFSP meal increased the actual proportion of this meal consumed. Specifically, those in the intention treatment indicated that the share on a plate of the OFSP meal they expected to eat would increase. Indication of this type of intention can be expected to direct thinking about realisation of the goal system, and to provide a plan for operationalising the goal-to-mean relationship. There may also be motivational consequences of thinking about the goal-to-mean relationship to the facilitation of action, because this process can help to anticipate how committed one is to pursuing the goal. This reduction in behavioural difficulty may increase the extent of mobilisation of available resources. The use of motivation as part of planning and behavioural maintenance to promote health-improving behaviour is well acknowledged in state-of-change models such as the Health Belief Model (Rosenstock, 1974). A limitation of the present study is that we only measured pre-intentions, so future research should examine how repetition of intentional behaviour can be used to sustain or re-direct commitment to goals. This step can provide a mechanism by which goal-to-mean structures are triggered into more long-lasting habits. By then combining attention to instrumental goals with repeated indication of intentions in a feedback loop approach, school meals programmes can be developed to better distinguish, and appropriately target, different stages in the change process of beneficiaries towards the desired direction.

Fourth, and in relation to behavioural maintenance, we found that use of an instrument to obtain repeated pairing of goals with the school meal (i.e. the stimulus) had no effect on the proportion of the OFSP meal eaten. This was unexpected, because the use of cards to repeatedly pair goals with the school meal was expected to counteract subjective effort or, alternatively, reinforce motivation to pursue the activity. This suggests that being reminded of one's own goals or using written goal-directed narratives may be rather ineffective in increasing consumption of a healthier food alternative. Therefore, a different approach to presenting messages about positive benefits may be needed when dealing with children.

Finally, the random error structure of the estimated models was largely driven by individual differences. Support for a combined group and individual effect on heterogeneity was only found in relation to the model for feelings after eating. This suggests that the statistical power could be increased by using (or adding) individually based covariates to reduce residual noise. There might be individual factors that vary both within and between groups of students. In most rural African communities, children often do not take part in food purchasing and preparation decisions, and hence their ability to eat a healthy diet may not really influence their actual behaviour. In addition, the food situation in most households of the study sample is likely to militate against nudging for healthy eating and promote eating habits/behaviours that are unhealthy or opportunistic. We hasten to add that these are speculative statements and that more analysis/research is needed to better determine how such individual or cultural components influence and predict behavioural outcomes.

## Conflicts of interest

The authors declare that they have no conflicts of interest with respect to authorship or publication of this article.

## Figures and Tables

**Table A1:** Characteristics of the school meal eaten and of the data per group of the study design.

	Group															
	1	2	3	4	5	6	7	8	9	10	11	12	13	14	15	16
OFSP meal eaten (g)	265	271	408	255	175	311	383	290	277	307	406	396	325	307	416	333
O-MEAL eaten (g)	166	231	180	114	152	123	191	160	219	160	157	139	229	165	161	261
Total food eaten (g)	431	489	588	369	327	434	574	450	496	467	563	535	554	472	577	594
Share of OFSP meal	0.61	0.55	0.69	0.69	0.54	0.72	0.67	0.64	0.56	0.66	0.72	0.74	0.59	0.65	0.72	0.56
Number of observations	127	203	125	186	113	196	87	256	118	184	63	219	118	248	115	182
Number of individuals	28	43	26	40	26	44	19	53	26	40	17	50	25	56	24	39

**Table A2:** Effect (logits) of study factors on proportion of the orange-fleshed sweetpotato (OFSP) meal eaten (n = 556, Nigeria).

Variable	Estimate	Std.Error	z-value	Pr(>|z|)
Intercept	3.79	0.71	5.36	<0.001
Goal generation^a^	0.67	0.53	1.27	0.20
Goal type^b^	1.73	0.71	2.44	0.015
Intentions stated^c^	0.94	0.38	2.47	0.013
Reminder used^d^	−0.03	0.39	−0.06	0.95
Time^e^ = 2	−0.91	0.43	−2.10	0.036
Time = 3	−0.69	0.44	−1.57	0.12
Time = 4	0.55	0.52	1.06	0.29
Time = 5	0.31	0.52	0.61	0.54
Goal generation (primed) × Goal type(experiential)	1.84	0.79	2.34	0.02
Goal type (instrumental) × time = 2	0.10	0.72	0.14	0.89
Goal type (instrumental) × time = 3	0.27	0.75	0.36	0.72
Goal type (instrumental) × time = 4	−2.15	0.77	−2.78	0.005
Goal type (instrumental) × time = 5	−0.52	0.86	−0.60	0.55
Random effects				
groups	name	variance		
id	intercept	7.198		

Number of obs.		2540		
AIC		851.4		
logLik		−411		
Overdispersion		0.65		

a Goal generation:1 = primed; 0 = self-generated; (M = 0.498).

b Goal type: 1 = instrumental; 0 = experiential; (M = 0.515).

c Intentions stated: 1 = intentions stated; 0 = no intentions revealed; (M = 0.485).

d Reminder used: 1 = card shown; 0 = no card shown; (M = 0.658).

e The time variable refers to the longitudinal aspect of the study (i.e. subsequent serving occasions for the OFSP meal).

**Table A3:** Effect (linear) of study factors on feelings after eating (n = 556, Nigeria).

Variable	Estimate	Std.Error	t-value	C.I. 2.5%|	C.I. 97.5%
Intercept	3.54	0.16	21.9	3.20	3.88
Goal generation	0.47	0.18	2.62	0.09	0.85
Goal type	−0.03	0.18	−0.18	−0.41	0.35
Intentions stated	−0.02	0.13	−0.17	−0.29	0.25
Reminder used	−0.15	0.13	−1.16	−0.42	0.12
Time = 2	0.27	0.05	4.89	0.16	0.37
Time = 3	0.30	0.06	5.41	0.19	0.40
Time = 4	0.45	0.06	8.02	0.34	0.56
Time = 5	0.47	0.06	8.00	0.35	0.58
Goal generation (primed) × Goal type(experiential)	0.280	0.26	1.08	−0.26	0.81
Random effects					
groups	name	variance			
Id:Group	intercept	0.21			
Group	intercept	0.05			
residual		0.76			

Number of obs.		2488			
AIC		6859.8			
logLik		−3416.9			

Note: C.I. = confidence interval. Coding for study factors as in Table A2.

**Table A4:** Effect sizes based on likelihood ratio tests of study factors on proportion of orange-fleshed sweetpotato (OFSP) meal eaten (n = 556, Nigeria).

Variable	Test	Proportion of OFSP meal eaten			
		df	AIC	logLik	p-value
0: Overall mean		2	868.0	−432.0	
A: Goal generation	0 vs A	3	860.2	−427.1	0.002
B: Goal type	A vs (A + B)	4	860.1	−426.1	0.152
C: Intentions stated	(A + B) vs (A + B + C)	5	856.3	−423.2	0.016
D: Reminder used	(A + B + C) vs (A + B + C + D)	6	858.1	−423.0	0.621
E: Goal generation (primed) × Goal type (experiential)	(A + B + C + D) vs (A + B + C + D + E)	14	862.5	−417.2	0.171
F: Goal type (instrumental) × time	(A + B + C + D + E) vs (A + B + C + D + E + F)	15	851.4	−410.7	0.0003

Note: Coding for study factors as in Table A2.

**Table A5:** Effect sizes based on likelihood ratio tests of study factors on feelings after the school meal (n = 556, Nigeria).

Variable	Test	Feelings			
		df	AIC	logLik	p-value
0: Overall mean		4	6941.6	−3466.8	
A: Goal generation	0 vs A	5	6932.2	−3461.1	<0.001
B: Goal type	A vs (A + B)	6	6932.7	−3460.4	0.23
C: Intentions stated	(A + B) vs (A + B + C)	7	6934.7	−3460.4	0.91
D: Reminder used	(A + B + C) vs (A + B + C + D)	8	6935.5	−3459.7	0.26
E: Time	(A + B + C + D) vs (A + B + C + D + E)	12	6858.9	−3417.4	<0.001
F: Goal generation (primed) × Goal type (experiential)	(A + B + C + D + E) vs (A + B + C + D + E + F)	13	6859.8	−3416.9	0.29

Note: Coding for study factors as in Table A2.

## Footnotes

Appendix B Supplementary data related to this article can be found at https://doi.org/10.1016/j.appet.2018.06.038.
