# Supplementary material for: Goal-setting and volitional behavioural change: Results from a school meals intervention with vitamin-A biofortified sweetpotato in Nigeria
Source: Appetite. 2018 Oct 1;129:113–24. doi: 10.1016/j.appet.2018.06.038 (PMC6102414; doi:10.1016/j.appet.2018.06.038)
Supplement: Multimedia component 2 [file mmc2.docx]

**Training session**

**The beginning**

**Note: The information was printed and presented on a big poster.**

**The enumerator will ensure that it is like a normal teaching session, allowing both chorus and individual responses etc from the pupils.**

**A: Five food groups:**

There are 5 broad groups of foods:

- Grains (cereal) like maize, roots like yam, sweetpotato
- Fruits & vegetables.
- Meat, poultry, fish, eggs, nuts and seeds and legumes/beans.
- Milk, yoghurt cheese and/or margarine.
- Fats, oils and sweets/candy.

**B: It is important to eat a balanced die**t,

Because not eating enough of the required quantities and types of food groups, consistently, will result in malnutrition (lack of energy and lack of important nutrients like vitamins) in the body

**C: The food pyramid**

The picture below summarizes the order of importance of various food groups in our diet:


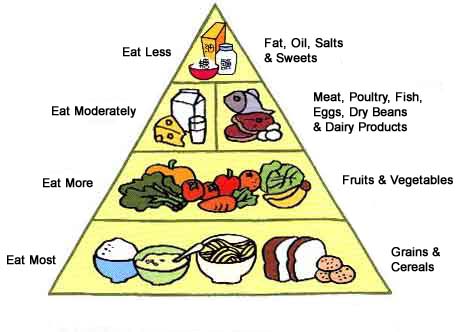


**Grains & Cereals, roots (group 1)** – give the body energy

**Fruits and vegetables (group 2)** – protect the body from diseases

**Protein (group 3 & 4)** – are body building foods

**Fats/Oils (group 5)** – are also energy giving foods

**D: A healthy food choice**

Healthy foods usually include fruits and vegetable and cereals that are high in fibre. A healthy food should have less fat, less refined starch. One should eat whole grains and meat that is not so fatty

**E: Incorporating OFSP in foods:**

We should also eat food containing vitamin A.

The figure below contains foods rich in vitamin A. Can you notice orange fleshed sweetpotatoes?


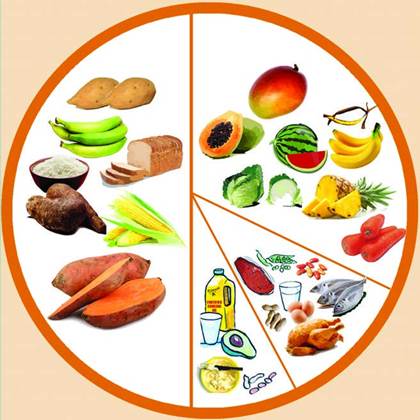


Food chart incorporating OFSP

Vitamin A is essential for good health. Vitamin A deficiency leads to blindness, and it makes somebody fall sick frequently. Children are among those at risk of Vitamin A deficiency. Crops such as OFSP are rich in Vitamin A (show the sweetpotato root). Sweetpotatoes can be recommended as a Vitamin A source.
